# Suppressor Analyses Identify Threonine as a Modulator of *ridA* Mutant Phenotypes in *Salmonella enterica*

**DOI:** 10.1371/journal.pone.0043082

**Published:** 2012-08-10

**Authors:** Melissa R. Christopherson, Jennifer A. Lambrecht, Deanna Downs, Diana M. Downs

**Affiliations:** Department of Bacteriology, University of Wisconsin–Madison, Madison, Wisconsin, United States of America; Indian Institute of Science, India

## Abstract

The RidA (YjgF/YER057c/UK114) family of proteins is broadly conserved in the three domains of life yet the functional understanding of these proteins is at an early stage. Physiological studies of *ridA* mutant strains of *Salmonella enterica* provided a framework to inform *in vitro* studies and led to the description of a conserved biochemical activity for this family. *ridA* mutant strains of *S. enterica* have characteristic phenotypes including new synthesis of thiamine biosynthetic intermediate phosphoribosylamine (PRA), inability to grow on pyruvate as a sole carbon and energy source or when serine is present in the minimal growth medium, and a decreased specific activity of transaminase B (IlvE). Secondary mutations restoring growth to a *ridA* mutant in the presence of serine were in *dapA* (encoding dihydrodipicolinate synthase) and *thrA* (encoding homoserine dehydrogenase). These mutations suppressed multiple *ridA* mutant phenotypes by increasing the synthesis of threonine. The ability of threonine to suppress the metabolic defects of a *ridA* mutant is discussed in the context of recent biochemical data and *in vivo* results presented here.

## Introduction

The RidA (formerly YjgF/YER057c/UK114) family of proteins is well conserved throughout the three domains of life. Members of this protein family have been implicated in a diverse number of phenotypes in a variety of organisms [1]–[12]. However, a common mechanism to explain these phenotypes was not obvious. Strains of *Salmonella enterica* lacking RidA display several characteristic phenotypes, including: synthesis of thiamine biosynthetic intermediate phosphoribosylamine (PRA), inability to grow on pyruvate as a sole carbon and energy source or in the presence of serine [13], and a decreased specific activity of transaminase B (IlvE) [14]. Each of these phenotypes required the presence of a functional threonine dehydratase (IlvA; EC 4.3.1.19). These phenotypic analyses in *Salmonella enterica* led to a general model in which RidA eliminated reactive products that were generated in normal metabolic reactions involving IlvA [5].

*In vitro* studies, which were informed by the phenotypic analyses, identified a biochemical function for the RidA protein family. RidA deaminated reactive enamine/imine metabolites generated by IlvA [15]. These enamine/imine compounds were normal intermediates in the pyridoxal-5′-phosphate-dependent dehydration of both threonine and serine. Further, reconstitution of the PRA formation phenotype required a short-lived intermediate produced by IlvA from threonine. This molecule, presumed to be the 2-aminocrotonate enamine was utilized by anthranilate phosphoribosyltransferase (TrpD; EC 2.4.2.18) to generate PRA [16]. RidA inhibited the formation of PRA *in vitro* by this mechanism, which was consistent with the phenotype observed only in a *ridA* mutant.

Aside from the IlvA-, TrpD-dependent formation of PRA, the *in vivo* consequences of a *ridA* mutation are not understood in the context of the biochemical activity of RidA. The *in vitro* biochemical work characterizing RidA did not address the significance of the enamine deaminase activity *in vivo* or relate the previously observed phenotypes to the *in vitro* activity. Herein suppressor analyses dissected the basis of the other phenotypes caused by the loss of RidA *in vivo*. The data showed that threonine reversed many of the phenotypes of a *ridA* mutant of *S. enterica*. We propose that threonine outcompetes serine for the active site of threonine dehydratase (IlvA) thus preventing the formation of a deleterious serine-derived reactive intermediate that is normally removed by RidA.

## Materials and Methods

### Bacterial Strains, Media, and Chemicals

Strains used in this study are derivatives of *S. enterica* serovar Typhimurium LT2 and are listed with their respective genotypes in Table 1.

**Table 1:** Bacterial strains.

Strain	Relevant Genotype*	Source
DM3480	*ridA3*::MudJ	Lab collection
DM3871	*ridA3*::MudJ *purF2085*	Lab collection
DM6309	*ridA3*::MudJ *purF2085 thrA1371*	This study
DM7608	*ridA3*::MudJ *ilvA3211*	[4]
DM7610	*ridA3*::MudJ *ilvA3210*	[4]
DM9404	Wild type (isogenic to DM3480)	Lab collection
DM9521	*ridA3*::MudJ *dapA356 zxx4116*::Tn*10*d(Tc)	This study
DM10009	*ridA3*::MudJ *ilvY3212*::Tn*10*d(Tc) *ilvA3210*	[4]
DM10010	*ridA3*::MudJ *ilvY3212*::Tn*10*d(Tc)	[4]
DM10331	*ilvY3212*::Tn*10*d(Tc) *ilvA3210*	[4]
DM10332	*ilvY3212*::Tn*10*d(Tc)	[4]
DM10460	*dapA362*::*cat*	This study
DM11412	*ridA3*::MudJ *purF2085 dapA356*	This study
DM11558	*ilvY3212*::Tn*10*d(Tc) *ilvA3211*	[4]
DM11609	*ridA3*::MudJ *thrA1371 stm0014-13*::Tn*10*d(Tc)	[4]
DM11635	*ridA3*::MudJ *dapA357*	This study
DM11636	*ridA3*::MudJ *dapA358*	This study
DM11637	*ridA3*::MudJ *dapA356*	This study
DM11638	*ridA3*::MudJ *dapA361*	This study
DM11639	*ridA3*::MudJ *dapA359*	This study
DM11640	*ridA3*::MudJ *dapA360*	This study
DM11877	*ridA3*::MudJ *thrA1371 stm0014-13*::Tn*10*d(Tc)	This study
DM11878	*ridA3*::MudJ *stm0014-13*::Tn*10*d(Tc)	This study
λ3520	Δ*asd*A1 *zhf4*::Tn*10*	R. Curtiss III [36]

* MudJ refers to Mud1734 [37]. Tn*10*d(Tc) refers to the transposition-defective mini-Tn*10*(Tn*10*Δ16Δ17 *tet^R^*) construct [38].

No-carbon E medium (NCE), supplemented with 1 mM MgSO_4_ [17], trace minerals [18], and 11 mM glucose (or 50 mM pyruvate as indicated) was used as minimal medium. Difco nutrient broth (8 g/L) with NaCl (5 g/L) was used as rich (NB) medium. Luria broth was used for experiments involving plasmid isolation. Super Broth containing tryptone (32 g/L), yeast extract (20 g/L), NaCl (5 g/L), and NaOH (5 mM) was used to grow cultures for protein purification. Difco BiTek agar was added (15 g/L) for solid medium. When present in the culture medium the final concentrations of serine and isoleucine were 5 and 0.3 mM, respectively. The final concentrations of the antibiotics in rich and minimal medium, respectively, were: tetracycline, 20, 10 mg/L, chloramphenicol, 20, 5 mg/L, and ampicillin, 150, 15 mg/L. Unless otherwise noted, all chemicals were from Sigma-Aldrich. Aspartate 4-semialdehyde was custom synthesized commercially at the University of Canterbury by the Gerrard Laboratory.

### Growth Quantification

Cells from overnight cultures in NB medium were pelleted and resuspended in an equal volume of saline (0.85% NaCl), and an aliquot (0.2 mL) was used to inoculate 5 mL of the appropriate minimal medium. Cell growth was monitored as optical density (OD) at 650 nm over time at 37°C with shaking. Growth rates (in h^−1^) were determined as µ =  ln(X/X_0_)/T where X =  OD at 650 nm and T =  time in hours during logarithmic growth.

### Genetic Techniques

Transductional crosses were performed using the high-frequency general transducing mutant of bacteriophage P22 (HT105/1, *int*-201) [19]. Methods for transductional crosses, purification from phage, and identification of phage-free transductants have been described elsewhere [20]. Multiply-mutant strains were constructed using standard genetic techniques. When necessary, genetic backcrosses were performed to confirm the presence of a respective allele.

To isolate mutants, independent cultures of *ridA3*::MudJ (DM3480) were grown overnight in NB, centrifuged, and resuspended in the same volume of saline. 10^7^ cells were spread on solid minimal glucose medium with 5 mM serine. Spontaneously arising mutations (∼10^−7^) that allowed *ridA* mutants to grow on serine were isolated after 36 hours at 37°C. A transposon (Tn*10d*(Tc)) genetically linked to the causative mutation in one strain was isolated by standard genetic techniques and used to reconstruct the mutant for phenotypic confirmation. The chromosomal location of relevant insertions was determined by sequencing using a PCR-based protocol [21]. A DNA product was amplified with degenerate primers and primers derived from the Tn*10d*(Tc) insertion sequence and sequenced at the University of Wisconsin Biotechnology Center. Strains carrying suppressor mutations were reconstructed by transducing the relevant allele into *dapA*::*cat* (DM10460) and selecting for growth without diaminopimelic acid.

### Molecular Techniques

The *dapA* genes from strains DM3480, DM7604, DM7606, and DM11019 were amplified by PCR using Herculase II Fusion DNA Polymerase (Stratagene) and primers 5′ DapANdeI (GGGGCATATGTTCACGGGAAGTATTC) and 3′DapAXhoI (GGGGCTCGAGTTACAGCAGGCCAGC) and cloned into the pET20b vector (Novagen) at *NdeI* and *XhoI* restriction sites. Sequence analysis of each clone confirmed the presence of the N-terminal hexahistidine tag and the relevant lesion. The construct carrying the wild-type allele (pLD-dapA) complemented a *dapA* mutant (DM10460), indicating that the gene was expressed in this construct (data not shown).

### Protein Purification

The wild-type and variant DapA proteins were overexpressed in *E. coli* BL21(AI) according to the manufacturer’s protocol (Invitrogen). Cells from the resulting cultures were broken at 15,000 psi in a French Pressure cell at 4°C. Cell debris was removed by centrifugation (42,000×*g*) for 30 min at 4°C. Proteins were purified using a column containing Ni-NTA superflow resin (QIAGEN) according to manufacturer’s protocol. Fractions containing DapA were concentrated at 30 psi under Argon gas using a 10,000 Da molecular weight cut-off membrane (Amicon). The protein was dialyzed in 0.5 M NaCl, 20 mM Tris-HCl, 5 mM imidazole, pH 7.9 and stored at −80°C. DapB was purified according to standard protocol using a hexahistidine-tagged *dapB* clone from the ASKA collection [22]. IlvE was purified as a hexahistidine-tagged protein as has been described [14]. Protein concentration was estimated with bovine serum albumin as the standard using a Bradford assay [23].

### Biochemical Assays

#### i) Dihydrodipicolinate synthase (DapA) assay

DapA activity was measured in a coupled assay with DapB (dihydrodipicolinate reductase; E.C. 1.3.1.26) following a published protocol [24]. A typical 1 mL reaction contained ∼2 µg DapB, 100 mM HEPES pH 8.0, 0.125 mM NADPH, 40 mM pyruvate, and 0.05–1.0 µg DapA (>95% pure) and was initiated by the addition of ASA at concentrations ranging from 0–2 mM. Enzyme-dependent oxidation of NADPH was quantified at 340 nm.

#### ii) Threonine dehydratase (IlvA) assay

IlvA was assayed as previously described [4], [25], or alternatively, by quantification of [^14^C]-2-ketobutyrate (2-KB) formed from [^14^C(U)]-L-threonine. 200 µL reactions containing 100 mM Tris pH 8.0, 50 µM pyridoxal-5′-phosphate, 20 mM ammonium chloride, 1 mM dithiothreitol (DTT), and 2 µg purified IlvA were initiated with a final concentration of 40 mM [^14^C(U)]-L-Threonine (12.5 µCi mmol^−1^), incubated for 12 minutes at 37°C, and stopped with 0.5 mL 0.1% 2,4-dinitrophenylhydrazine in 2 N HCl. Derivatized [^14^C]-2-KB was extracted with 0.5 mL toluene and radioactivity from 200 µL toluene phase, representing quantity of [^14^C]-2-KB generated, was counted in 5 mL scintillation fluid using a scintillation counter (Packard).

#### iii) Transaminase B (IlvE) assay

The transaminase B activity assay was based on previously described protocols [14], [26]. Cells were permeabilized by sonication. Known concentrations of product 2-keto-3-methylvalerate were subjected to the extraction procedure to generate a standard curve.

#### iv) Homoserine dehydrogenase (ThrA) assay

The homoserine dehydrogenase activity assay was adapted from a previously described protocol [27]. Cells were grown in 100 mL minimal medium to an OD_650 nm_ of 0.8, pelleted, and resuspended in 0.5 mL 100 mM HEPES pH 8.0 with 0.125 mM DTT. Cells were disrupted by sonication, extract was clarified by centrifugation, and total protein concentration was estimated by the method of Bradford [23]. Assay mixtures contained 100 mM HEPES pH 8.0, 0.125 mM DTT, 200 mM potassium chloride, 0.3 mM NADP+, and ∼300 µg cell extract, in a final volume of 200 µL. Assays were initiated by the addition of 15 mM homoserine and activity was monitored by the increase in absorbance at 340 nm at 30°C, representing NADPH production. Inhibitor L-threonine was added to a final concentration of 0.5 mM when indicated.

## Results

### Alleles of *dapA* Restore Growth of a *ridA* Mutant Strain on Glucose Serine

**Figure 1:**
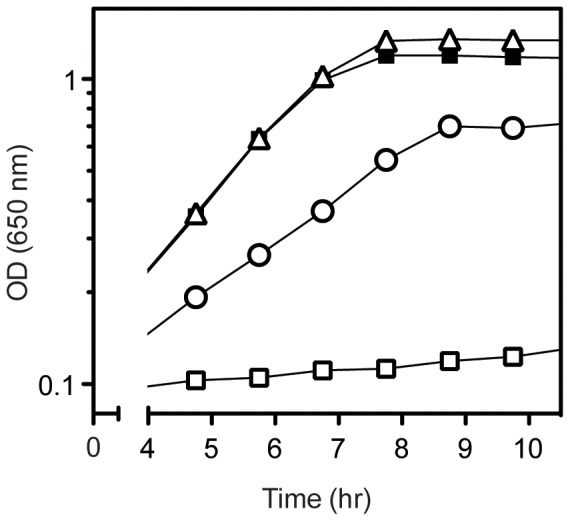
Mutations in *dapA* restore growth to *ridA* mutants in the presence of serine. Growth was monitored over time as optical density at 650 nm. Strains were grown at 37°C in minimal glucose medium with no additions (closed symbols) or 5 mM serine (open symbols). Shown are strains *ridA* (DM3480), squares; *ridA dapA356* (DM11637), triangles; and *ridA dapA360* (DM11640), circles. Curves displayed were representative of 3 biological replicates.

A *ridA* null mutant (DM3480) cannot grow on minimal glucose medium in the presence of 5 mM serine [13]. Six independent mutant derivatives of *ridA* that grew in the presence of serine were isolated. Using Tn*10*d(Tc) insertions to map the location of the mutations, each of the causative mutations was subsequently found to affect the *dapA* locus, encoding dihydrodipicolinate synthase (EC 4.2.1.52). Table 2 summarizes the six lesions that allowed growth of the *ridA* mutant in the presence of serine. Four lesions generated variant DapA proteins (DapA_A563G_ was isolated twice), one affected the Shine-Dalgarno sequence and one was in the *dapA* promoter. Strains with each of the mutant alleles were reconstructed (DM11635–40) and were analyzed in liquid media for growth in the presence of serine. *ridA* mutant strains containing alleles *dapA356, dapA357,* or *dapA358* grew similar to a wild-type strain in the presence of serine and are represented by strain DM11637 in Figure 1. The parent *ridA* strain (DM3480) failed to grow after 12 hours as expected. The strain carrying a lesion 36 nucleotides upstream of *dapA* (DM11640) had limited growth with serine and was concluded to decrease transcription of the *dapA* gene. (The promoter of *dapA* from *E. coli* resides within a 70-base region upstream of *dapA* containing an extended −10 and −35 site [28].) Growth of the suppressor-containing strains, with the exception of strain *ridA dapA359* (DM11639), was indistinguishable from the parental strain on minimal glucose medium (data not shown). The *dapA359* allele encoded a variant with two deleted amino acid residues and despite growth on solid medium with serine, growth was not detected in liquid media after 24 hours in the absence of exogenous diaminopimelic acid (DAP).

**Table 2:** Suppressing DapA variants have decreased specific activities.

Strain	Allele*	DNA change	Proteinchange	Specificactivity†
DM9404	WT	–	–	5.10±1.60
DM11637	*dapA356*	A563G	D188G	0.12±0.04
DM11635	*dapA357*	C143T	S48F	0.15±0.04
DM11636	*dapA358*	A(−10)T	–	N.D.‡
DM11637	*dapA359*	ΔG249–C254	ΔE84–A85	0.02± <0.01
DM11640	*dapA360*	T(−36)C	–	N.D.
DM11638	*dapA361*	A563G	D188G	N.D.

* A *ridA* strain carrying any of the listed alleles is able to grow in the presence of serine.

† Specific activity of DapA in µmol NADPH oxidized/sec/mg of purified protein.

‡ N.D.  =  not determined.

### Suppressor Alleles of *dapA* Encode Variants with Decreased Specific Activity

The wild-type gene and each of three suppressor alleles of *dapA* were cloned into the pET20b vector to generate C-terminal hexahistidine tagged proteins, creating pLD-dapA, pLD-dapA_D188G_, pLD-dapA_S48F_ and pLD-dapA_Δ84–85_. The recombinant proteins were purified by affinity chromatography. Wild-type and variant proteins were assayed for dihydrodipicolinate synthase activity using a coupled assay [24]. The variant proteins all had more than a 30-fold decrease in specific activity when compared to the wild-type protein, as shown in Table 2.

A simple interpretation of the above results was that decreased activity of DapA allowed growth of a *ridA* mutant in the presence of serine. Complementation analysis eliminated the formal possibility that an altered function of DapA was responsible for allowing growth of a *ridA* mutant. When provided *in trans*, wild-type *dapA* eliminated growth of the *ridA dapA356* mutant strain in the presence of serine and did not affect growth of a *ridA* mutant (data not shown).

### Aspartate 4-semialdehyde Accumulation Mediated Phenotypic Suppression by the *dapA* Alleles

**Figure 2:**
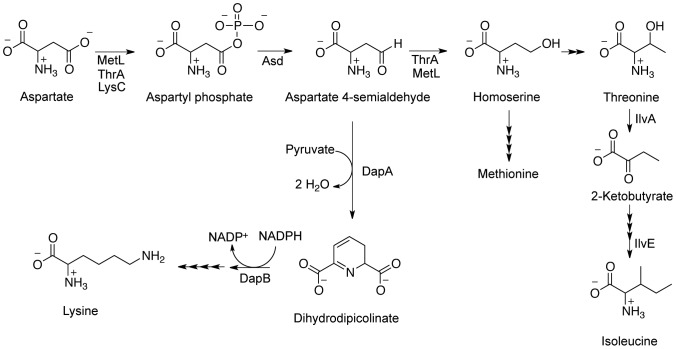
Pathway for synthesis of aspartate-derived amino acids. Aspartate is a precursor to lysine, methionine, threonine, and isoleucine, as depicted here. Aspartate 4-semialdehyde (ASA) is a branchpoint metabolite controlled by the activities of DapA, ThrA, and MetL.

DapA functions in the synthesis of some aspartate-derived amino acids and uses aspartate 4-semialdehyde (ASA) as a substrate (Figure 2). In one scenario, a recessive lesion in *dapA* results in accumulation of ASA that allows a *ridA* mutant to grow in the presence of serine. ASA itself restored the growth of a *ridA* mutant in the presence of serine, supporting a role for this molecule in suppression of the *ridA* phenotype. As little as 0.5 mM ASA in the medium allowed a *ridA* mutant to reach full density in medium with 5 mM serine. Growth rate (µ) of *ridA* (DM3480) in the presence of serine (µ = 0.06±0.01) was restored by 1 mM ASA (µ = 0.55±0.03) and was the same as the growth rate of the same strain grown on minimal medium without serine (µ = 0.54±0.03). The nutritional requirements of an *asd* mutant (methionine, lysine, DAP, and threonine), which cannot make ASA, [29] were satisfied with ∼1.3 mM exogenous ASA, indicating the cells have the ability to transport and incorporate ASA into the biosynthetic pathways (data not shown).

In addition to suppressing serine sensitivity, the *dapA* alleles restored IlvE activity in a *ridA* mutant. The IlvE activity in the *ridA* strain carrying the *dapA356* allele (230±7 nmol/min/mg) was restored to an intermediate level between the wild-type (303±13 nmol/min/mg) and *ridA* mutant strain (140±7 nmol/min/mg). This result suggested intracellular accumulation of ASA could impact the activity of IlvE in a *ridA* mutant. No evidence of a direct role for ASA in mediating phenotypic suppression was found. The activity of purified IlvE was not significantly affected by 10 min incubation with 10 mM ASA (26.1±7 µmol/min/mg without ASA, 18.6±6 µmol/min/mg with ASA). Further, ASA had no detectable effect on the activity of threonine deaminase (IlvA) *in vitro.* While as little as 500 µM isoleucine inhibited IlvA, ASA failed to inhibit IlvA *in vitro* at a range of concentrations (0.1 µM –1.0 mM) (data not shown). These data showed that the effect of ASA was not due to mimicking the effect of isoleucine as a feedback inhibitor [14], and suggest that further metabolism of this molecule was required.

### Analysis of a Second Suppressor Locus Provides Insight into Role of ASA

In addition to the alleles of *dapA* described above, a mutation in *thrA* (*thrA1371*), encoding aspartokinase I/homoserine dehydrogenase I, previously reported to suppress serine sensitivity of a *ridA* mutant [4] was sequenced and found to encode variant ThrA_G403D_. The homoserine dehydrogenase activity in a strain with the ThrA_G403D_ variant was indistinguishable from the wild-type parental strain. The location of the G403D substitution suggested the variant could be altered in allosteric interaction properties [30]–[32]. Data in Table 3 showed that the homoserine dehydrogenase activity of the ThrA_G403D_ variant was resistant to inhibition by threonine. Significantly, this effect was evident at a low of concentration of threonine, as would be expected under *in vivo* conditions where the threonine concentration was reported to be 0.2 mM [33]. Taken together, the data suggested the ThrA_G403D_ variant could increase conversion of ASA to homoserine *in vivo*, consistent with the above conclusion that metabolism of ASA is required for suppression.

**Table 3:** The ThrA_G403D_ variant is insensitive to feedback inhibition by threonine and serine.

		Homoserine dehydrogenaseactivity*	
*thrA* allele	Protein variant	No inhibitor	+ Thr (0.5 mM)
*thrA* WT	WT	44±5	18±3
*thrA1371*	ThrA_G403D_	37±5	38±6

* Homoserine dehydrogenase activity was measured in crude extracts from isogenic strains DM11877 (*ridA thrA1371*) and DM11878 (*ridA*) by following reduction of NADP+ and was reported as ΔA_420 nm_/min/µg protein.

### Threonine, not Isoleucine is the Metabolite Responsible for Suppression

ASA is a biosynthetic precursor to isoleucine, which is known to allow a *ridA* mutant to grow in the presence of serine [13], so it was a formal possibility that ASA was correcting growth by leading to increased levels of isoleucine. Two IlvA variants with decreased threonine dehydratase activity were used to constrict flux between ASA and isoleucine. Neither of the *ilvA* alleles caused a detectable growth defect on minimal glucose medium (Table 4). However, they each resulted in derepression of the *ilv* operon [4] indicating the strains were limited for isoleucine. Despite the constriction of flux between ASA and isoleucine, the double mutants *ridA ilvA3210* (DM10009) and *ridA ilvA3211* (DM11558) had the same growth rates as a *ridA* mutant (DM10010) (µ = 0.53±0.10, 0.54±0.04, and 0.56±0.01, respectively) when grown in a minimal medium containing 5 mM serine and 1 mM ASA. These data suggested that ASA did not correct growth by increasing intracellular isoleucine levels.

**Table 4:** IlvA variants have reduced activity.

ilvA allele	Proteinvariant	Activity*	µ†(Glc)	µ†(Glc Ile)
ilvA WT	WT	0.22±0.01	0.54±0.05	0.53±0.01
ilvA3210	IlvA_A142T_	B.D.‡	0.62±0.01	0.60±0.03
ilvA3211	IlvA_G191S_	0.05±0.01	0.56±0.03	0.54±0.01

* Threonine dehydratase (IlvA) activity measured in crude extracts from DM3480 (*ridA*), DM7610 (*ridA ilvA3210*) and DM7608 (*ridA ilvA3211*) and reported as ΔA_540 nm_/min/mg protein.

† Growth rate (in h^−1^) (µ =  ln(X/X0)/T where X =  optical density at 650 nm and T =  time in hours during logarithmic growth) for strains DM10332 (WT), DM10331 (*ilvA3210*), and DM11558 (*ilvA3211*) determined from growth in minimal medium with glucose (Glc) and glucose with isoleucine (Glc Ile).

‡ Below Detection.

Other metabolites in the pathway from ASA to the branch chain amino acids were considered and tested for their ability to suppress growth of a *ridA* mutant with serine. Nutritional tests showed qualitative suppression of multiple phenotypes with both homoserine and threonine. Addition of exogenous threonine to the growth medium of a *ridA* mutant restored growth on serine (µ = 0.09±0.01 without threonine, 0.50±0.01 with threonine), growth on pyruvate (µ = 0.06±0.01 without threonine, 0.37±0.02 with threonine), and IlvE activity (160±31 nmol/min/mg in minimal medium without threonine versus 287±33 nmol/min/mg in minimal with threonine).

**Figure 3:**
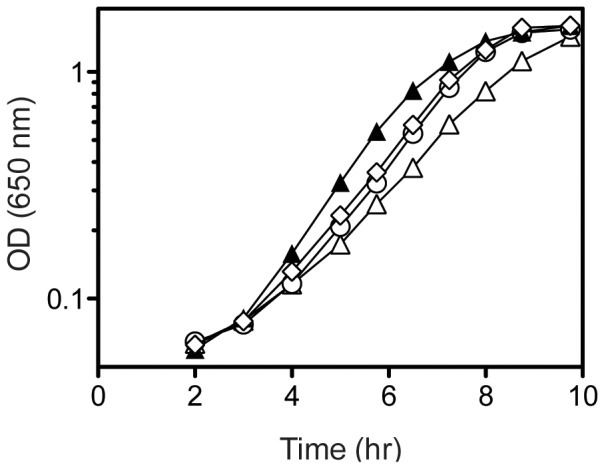
Suppressor mutations increase growth in *purF ridA* strain background. Strains were grown at 37°C in minimal glucose medium with adenine (open symbols) or further supplemented with 0.3 mM threonine (closed symbols). Growth was monitored over time as optical density at 650 nm. Shown are strains *purF ridA* (DM3871), triangles; *purF ridA thrA1371* (DM6309), diamonds; and *purF ridA dapA356* (DM11412), circles. Error bars represent standard deviations of three biological replicates.

Threonine is a precursor in PRA formation in a *ridA* mutant [16]. This fact provided a means to directly test whether the suppressor mutations in *dapA* and *thrA* generated increased cellular threonine levels. If the *dapA* and *thrA* mutations acted by increasing flux to threonine, they would be expected to increase the PRA formed in a *ridA* mutant. A *purF* mutant strain background was used to detect PRA, as it requires PRA to make thiamine and allow growth. The data in Figure 3 showed that the *thrA* and *dapA* suppressors increased growth of a *purF ridA* strain, and exogenous threonine further increased growth. These results supported the conclusion that flux to threonine was increased by these mutations. Additionally, since isoleucine has been shown to have the opposite effect and inhibit PRA synthesis in a *ridA* mutant [13], these data were consistent with the interpretation that the *dapA* mutations were not increasing the synthesis of isoleucine. Considering the results of nutritional and suppressor analyses in total, threonine was identified as the metabolite that had a direct effect in suppressing the phenotypes caused by lack of RidA.

## Discussion

The RidA (YjgF/YER057c/UK114) family of proteins is highly conserved, but the diverse cellular defects caused by its absence are not understood [1]–[11]. Recently it was shown *in vitro* that RidA family members deaminate reactive enamine/imine intermediates generated by threonine dehydratases (*e.g*., IlvA) [15]. This study investigated the relationship between the characterized biochemical activity of RidA and the *in vivo* phenotypes observed in a *ridA* mutant in *S. enterica*. Suppressor analyses identified an important role for threonine in attenuating multiple phenotypes of a *ridA* strain, including sensitivity to exogenous serine, lack of growth on pyruvate, and a decreased specific activity of IlvE.

When considering the results of this study in combination with the biochemical activity of RidA, we proposed a mechanism by which threonine could suppress the mutant phenotypes. Our model predicted that threonine relieved the sensitivities of a *ridA* mutant by outcompeting serine in the IlvA active site. Threonine dehydratase (IlvA) was required for a number of *ridA* phenotypes [4], [13], [14], [16]. The fact that threonine reversed those phenotypes suggested the metabolic defects required IlvA to use a different substrate. To our knowledge, the only other reported physiological substrate of IlvA is serine, and IlvA has a much higher *K_m_* for serine than for threonine (90 mM versus 4.5 mM, respectively [34]). Threonine and serine use the same active site in IlvA [35], and the presence of additional threonine would preclude IlvA from binding and dehydrating serine instead. This model suggested that the intermediate derived from serine, but not threonine, was deleterious to the cell unless it was removed by RidA.

The significance of threonine as a key metabolite that can modulate the *ridA* serine-sensitivity phenotype was further emphasized by the saturation of the suppressor analyses. Repeated attempts to isolate serine-resistant mutants only produced the decreased activity *dapA* (dihydrodipicolinate synthase) alleles and the feedback-resistant *thrA* (homoserine dehydrogenase) allele described here. These mutants not only demonstrated that increased flux to threonine was key to reversing the serine-sensitivity of a *ridA* mutant, but they also suggested that the primary control of threonine levels in the cell occurs at the homoserine dehydrogenase step and can be affected by increasing substrate (ASA) or decreasing the allosteric control of ThrA. This finding has important implications for metabolic engineering and groups endeavoring to generate organisms that overproduce threonine or downstream metabolites.

The findings herein emphasized the central role of threonine in compensating for the lack of RidA. In combination with past results, these data refine a model to explain the phenotypes of *ridA* mutants. It has been shown that IlvA generates reactive enamine/imines that are removed by RidA [15]. We suggest that serine is used as a substrate by IlvA to generate a reactive intermediate that attacks cellular components if it is not quenched by RidA. This is in contrast to the reactive intermediate derived from threonine reported to serve as a substrate for an alternative mechanism of PRA synthesis [16]. Thus, the IlvA-generated intermediates that accumulate *in vivo* in the absence of RidA can have either deleterious or productive consequences, depending on the substrate used (*e.g.,*serine versus threonine). Together these results suggest a complex role for IlvA in the *in vivo* phenotypes of *ridA* mutants. Continued studies are needed to identify the diversity of both the reactive metabolites eliminated by RidA and the targets of these reactive intermediates to better understand the breadth of metabolic consequences that result from the lack of the conserved RidA protein.
